# Associations between Polycystic Ovary Syndrome (PCOS) and Antibiotic Use: Results from the UAEHFS

**DOI:** 10.3390/antibiotics13050397

**Published:** 2024-04-26

**Authors:** Nirmin F. Juber, Abdishakur Abdulle, Amar Ahmad, Fatme AlAnouti, Tom Loney, Youssef Idaghdour, Yvonne Valles, Raghib Ali

**Affiliations:** 1 Public Health Research Center, New York University Abu Dhabi, Abu Dhabi P.O. Box 129188, United Arab Emirates; aa192@nyu.edu (A.A.); asa12@nyu.edu (A.A.); yi3@nyu.edu (Y.I.); yv8@nyu.edu (Y.V.); ra107@nyu.edu (R.A.); 2 College of Natural and Health Sciences, Zayed University, Abu Dhabi P.O. Box 19282, United Arab Emirates; fatme.alanouti@zu.ac.ae; 3 College of Medicine, Mohammed Bin Rashid University of Medicine and Health Sciences, Dubai Health, Dubai P.O. Box 505055, United Arab Emirates; tom.loney@mbru.ac.ae; 4 MRC Epidemiology Unit, University of Cambridge, Cambridge CB2 0SL, UK

**Keywords:** antibiotic use, polycystic ovary syndrome, PCOS, global health, epidemiology

## Abstract

Women with polycystic ovary syndrome (PCOS) have a higher susceptibility to infections compared to those without PCOS. Studies evaluating antibiotic use based on PCOS status are scarce. Therefore, we aimed to (i) assess the associations between self-reported PCOS and antibiotic use, and (ii) whether PCOS treatment and the age at PCOS diagnosis modified the associations above. This cross-sectional analysis used the United Arab Emirates Healthy Future Study (UAEHFS) conducted from February 2016 to March 2023 involving 2063 Emirati women aged 18–62 years. We performed ordinal logistic regressions under unadjusted and demographic-health-characteristic-adjusted models to obtain the odds ratios (ORs) and 95% confidence intervals (CIs) to analyze PCOS and antibiotic use. Subgroup analyses were performed by treatment status and age at diagnosis. We found that women with PCOS were 55% more likely to frequently take a course of antibiotics in the past year (aOR 1.55; 95% CI 1.26–1.90). Similar likelihoods were also found among those being treated for PCOS and those without treatment but with a PCOS diagnosis at ≤25 years. Our study suggests that PCOS was associated with an increased use of antibiotics among Emirati women. Understanding the frequent antibiotic use susceptibility among those with PCOS may improve antibiotic use surveillance and promote antibiotic stewardship in these at-risk individuals.

## 1. Introduction

Polycystic ovary syndrome (PCOS) is a common endocrine disorder affecting 4–20% of women of reproductive age worldwide, depending on the diagnostic criteria used [1,2]. The etiology of PCOS is not exactly known; however, adiposity, ovarian follicle development, insulin sensitivity, and chronic systemic inflammation have been proposed as a few possible mechanisms [3]. The common features of PCOS include irregular menstrual cycles, elevated testosterone levels leading to a hormonal imbalance or hyperandrogenism, and metabolic disorders [4,5,6]. PCOS is a multifactorial disorder with known risk factors including genetic predisposition, dietary factors or nutritional status, and obesity [6,7,8,9]. PCOS has been recognized as a chronic metabolic disorder, not just an endocrine disorder, due to the metabolic disturbances that accompany PCOS [10]. The consequences of this metabolic disturbance are that women with PCOS are often burdened with multiple chronic morbidities and multiple medications to treat their conditions [11,12]. The treatment of PCOS usually involves multimodal or combination approaches due to the complex etiology of PCOS, depending on the prevailing PCOS symptoms [9]. Several factors in PCOS treatment need to be considered in treating PCOS effectively, including the gut microbiota composition, as gut dysbiosis is known to be one of the risk factors for PCOS [9,13,14].

Antibiotic use is a major public health concern as the inappropriate use of antibiotics leads to antibiotic resistance, which significantly threatens human health [15]. In 2019, the World Health Organization considered antibiotic resistance as one of the top ten threats to global health [16]. Several factors have been known to be associated with the increase in antibiotic resistance, including excessive antibiotic prescriptions by medical professionals as well as self-prescription or self-medication [17,18,19]. Frequent antibiotic use has been associated with increased disease risks, such as colorectal cancer risk and second breast cancer events [20,21]. Antibiotics are essential in treating life-threatening conditions caused by bacteria. With the appropriate and effective use of antibiotics, the successful treatment of bacterial infections can be achieved and will improve human health [22]. Therefore, the close surveillance or monitoring of at-risk individuals for increased antibiotic use may improve the effectiveness of antibiotics in the population.

PCOS is a pro-inflammatory state and the endocrine–immune features of this disorder may explain the link between PCOS and infections, hence the antibiotic use [23]. Previous studies reported that women with PCOS had a higher susceptibility to various infections than those without PCOS [11,24,25]. Furthermore, women with PCOS may exhibit obesity [4], and the frequent co-occurrence of obesity with PCOS has been observed [26]. This may lead to increased antibiotic consumption among women diagnosed with PCOS, particularly obese women with PCOS. Studies have shown that a compromised immune response and leptin deficiency in obese individuals were found to be associated with an increased susceptibility to infections [27,28].

To date, there are limited population studies aimed at revealing the link between PCOS status and antibiotic use. To our knowledge, there is no study evaluating the association between PCOS and antibiotic use considering PCOS treatment and PCOS phenotypes, such as PCOS in adolescents and adults. In addition, the roles of comorbidity and obesity in the associations between PCOS and antibiotic use have not been widely discussed. Therefore, we aimed to investigate the associations between PCOS status and antibiotic use with further stratifications by PCOS treatment status and the age at PCOS diagnosis. Further, we performed sensitivity analyses to better reveal the roles of comorbidity and obesity in the associations between PCOS-related status and antibiotic use.

## 2. Material and Methods

We investigated the associations of PCOS-related status—namely women with PCOS versus those without PCOS, women being treated with PCOS versus those without treatment, and women diagnosed with PCOS earlier in life (at the age of 25 years or younger) versus those diagnosed with PCOS later in life (after the age of 25)—with antibiotic use in the past year.

### 2.1. Study Design, Participants, and Setting

**Figure 1:**
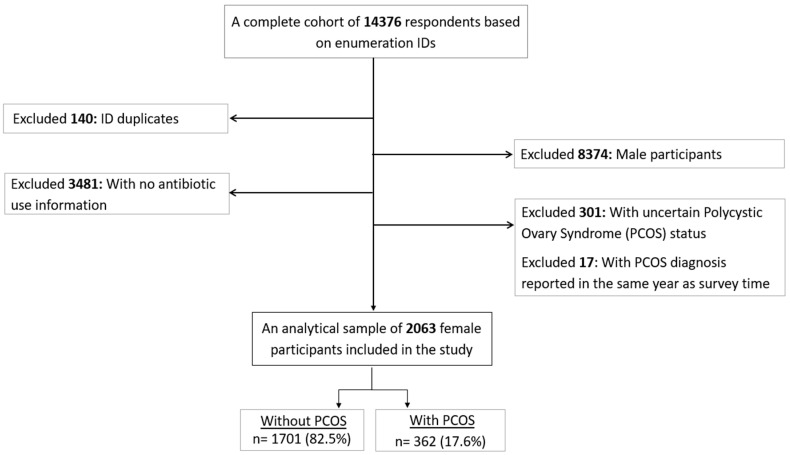
Flowchart of the final analytical sample included in the study.

This was a cross-sectional analysis of the United Arab Emirates Healthy Future Study (UAEHFS) conducted from February 2016 to March 2023. From a complete cohort of 14,376 individuals based on numeration IDs in the UAEHFS, we included 2063 Emirati women aged 18–62 years who provided complete information on PCOS status and antibiotic use in the past year; a complete case method (Figure 1). We excluded women with uncertain responses on PCOS status and antibiotic use in the past year (do not know, prefer not to answer, and missing). The study design and methodology of the UAEHFS are described elsewhere [29]. In brief, the UAEHFS is a population-based multirecruitment center study that recruited Emirati adults aged 18 years or above across the UAE. Participants were asked to fill out the online questionnaire and had some physical measurements assessed in the participating centers. Women who participated in this study were asked whether they were pregnant at the survey time, and only non-pregnant women were recruited into the cohort. Due to the COVID-19 pandemic, the recruitment shifted to being online-based starting in April 2020.

### 2.2. Measurements

#### 2.2.1. Antibiotic Use as an Outcome Variable

We analyzed self-reported antibiotic use in the past year based on the questionnaire response to “How often have you taken a course of antibiotics in the last year?” (never, once, twice, or three times or more) [30]. We treated antibiotic use as an ordinal outcome due to the ordered categories of the questionnaire responses.

#### 2.2.2. PCOS-Related Status as Exposure Variables

We analyzed self-reported diagnosis of PCOS based on the questionnaire response to: “Has a doctor ever told you that you have polycystic ovarian syndrome or disease?” (yes or no). The self-reported age at PCOS diagnosis was extracted from the questionnaire response to “How old were you when the doctor first told you that you had polycystic ovarian syndrome or disease?”. We then used the age of 25 years as a cut-off for PCOS diagnosis categorization, similar to previous epidemiological studies [12,31,32]. Lastly, PCOS treatment status was extracted from the questionnaire item “Are you being treated for polycystic ovarian syndrome or disease?” (yes or no).

#### 2.2.3. Demographic Characteristics and Health Profiles as Third Variables

The questionnaire responses determined age (years in continuous form). Urbanicity was determined based on the questionnaire response to “Where do you and your family live now?” (urban or rural/non-urban areas). Education levels were constructed based on the questionnaire response to “What is the highest level of education that you have completed?”. We then categorized education levels into two categories: ≤12 years and >12 years of schooling [33]. Body mass index (BMI) was calculated using the Tanita MC 780 by nurses at the recruitment centers, and we categorized the BMI levels into normal or low BMI (<25 kg/m^2^), overweight (25 to <30 kg/m^2^), and obese (≥30 kg/m^2^) [34]. Overall health was determined based on the questionnaire response to “In general how would you rate your overall health now?” (poor, fair, good, or excellent). We then categorized overall health status into poor/fair or good/excellent categories based on the responses [35]. The smoking variable was determined based on the questionnaire response to “Have you ever smoked cigarettes, even one time?” (yes or no). Next, regular medication use was determined based on the questionnaire responses to the item “Do you regularly take any of the following high blood sugar medication/high cholesterol medication/high blood pressure medication/aspirin/paracetamol/regular prescription medication/vitamins/other supplementations?”. We then categorized regular medication use into yes (if at least one previously stated medication was being reported) or no (if no medication was being reported). Lastly, the comorbidity in this study was determined based on the questionnaire response to “Has a doctor ever told you that you had [disease name]?” (yes or no). We included three pro-inflammatory chronic conditions, namely diabetes, high cholesterol, and high blood pressure. Those with comorbidity were defined as individuals who reported at least one of the above-mentioned chronic conditions at the survey time.

### 2.3. Statistical Analysis

Demographic characteristics and health profiles of study participants based on their PCOS status were evaluated using medians with interquartile ranges (median, IQR) for continuous variables and frequencies with percentages (*n*, %) for categorical variables. Distributions of antibiotic use in the past year based on PCOS status, as well as by PCOS treatment status and the age at PCOS diagnosis, were evaluated using frequencies with percentages (*n*, %). We further tested the distribution of our data for the parallel lines assumption for an ordered logistic regression. Since the assumption was met for our data, we then performed ordinal logistic regressions to estimate the odds ratios (ORs) and 95% confidence interval (CI), respectively, for the associations between PCOS status and antibiotic use in the past year among the total participants (main analysis), as well as by PCOS treatment and the age at PCOS diagnosis (stratification analysis). Women without PCOS history were used as a reference group in any analyses. We examined the ORs under two models: unadjusted and adjusted models. The complete case method was used to handle missing values in the regression analyses. We adjusted for current demographic characteristics and health profiles based on the literature and those found in the dataset, namely age [36], urbanicity [37,38], education level [39,40], BMI [12,41], overall health [36], smoking [35,42], and regular medication use [36], as these variables are known to be associated with PCOS and antibiotic use based on the literature. All analyses were performed using STATA 17.0 (StataCorp, College Station, TX, USA). *p*-values of <0.05 were considered statistically significant.

### 2.4. Ethical Approval

The study and its procedures have been reviewed and approved by the Institutional Review Board at New York University Abu Dhabi, Dubai Health Authority, Ministry of Health and Prevention in the UAE, and Abu Dhabi Health Research and Technology Committee, with the reference number of DOH/HQD/2020/516. Written consent was obtained from participants at the participating recruitment centers or by filling out an online consent form before data collection started.

## 3. Results

The demographic characteristics and health profiles of the study participants based on PCOS status are shown in Table 1. Compared to women without PCOS, those with PCOS were older (28.5 ± 8.1 vs. 22.0 ± 8.1 years) and a greater proportion resided in urban areas (90.1% vs. 85.9%), had higher education levels or >12 years of schooling (65.7% vs. 53.9%), had a higher BMI (26.6 ± 6.6 vs. 23.9 ± 6.6 kg/m^2^), were in poor or fair health (27.9% vs. 25.0%), reported having ever smoked (13.3% vs. 8.7%), and reported regular medication use (68.2% vs. 58.7%).

**Figure 2:**
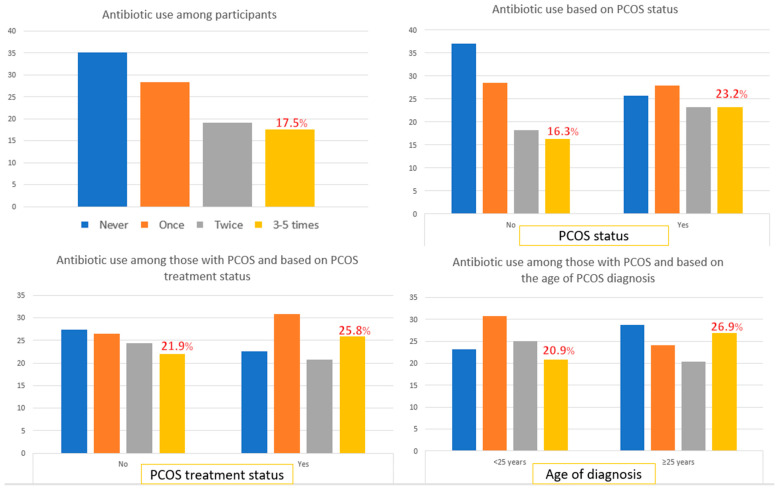
Antibiotic use in the past year among study participants, based on PCOS-related status.

In addition, the proportion of antibiotics used in the past year among study participants based on PCOS status, PCOS treatment status, and the age at PCOS diagnosis are shown in Figure 2. A third of participants reported never having a course of antibiotics in the past year. Meanwhile, 17% reported having two or more antibiotic courses in the past year (frequent users). In PCOS status stratification, more women with PCOS were reported as frequent antibiotic users compared to women without PCOS (23.2% vs. 16.3%). Women being treated for PCOS as well as those with PCOS diagnosed at ≥25 years were more likely to report being frequent antibiotic users compared to their respective counterparts (25.8% versus 21.9% in the PCOS treatment group and 26.9% versus 20.9% in the age at PCOS diagnosis group).

The associations between PCOS status and antibiotic use in the past year among participants (main analysis), as well as PCOS treatment status and the age at PCOS diagnosis (stratified analyses), are presented in Table 2. Women with PCOS were 55% more likely to frequently take a course of antibiotics in the past year compared to women without PCOS. Similar likelihoods were also observed in all stratified analyses, except in the ≥25 years at PCOS diagnosis group. The strongest association between PCOS and antibiotic use was observed among women being treated for PCOS, compared to those without PCOS, history after adjusting for confounding factors (aOR = 1.70; 95% CI: 1.22–2.38).

Similar regression analysis strategies restricted only among those reported comorbidities are highlighted in Table 3. In all adjusted models, the previously observed significant association between PCOS and antibiotic use in the past year persisted, even after considering the comorbid conditions. In this comorbidity-stratified analysis and referring to the main regression analysis, the greatest increase in the magnitude of the associations between PCOS and antibiotic use was observed among women with PCOS diagnosed at ≤25 years of age compared to those without PCOS history (aOR = 2.09; 95% CI: 1.30–3.36).

Lastly, a restricted analysis to assess the possibility of whether obesity mediates the associations between PCOS-related status and antibiotic use are presented in Table 4. This table shows the ORs for the associations between PCOS status and antibiotic use in the past year, restricted only among obese participants, in the main analysis and stratified analyses by PCOS treatment status and the age at PCOS diagnosis. All statistically significant associations found in the previous analyses disappeared if the analysis was restricted to only obese women with PCOS versus obese participants without PCOS. In this obesity-stratified analysis and referring to the main regression analysis, interestingly, the greatest decrease in the magnitude of the associations between PCOS and antibiotic use was observed among women being treated for PCOS, compared to those without PCOS history (aOR = 1.19; 95% CI: 0.57–2.46).

## 4. Discussion

The crude prevalence of self-reported PCOS in this cross-sectional study was 17.6% (Table 1). The prevalence in this study is similar to a UAE study involving Emiratis and non-Emirati nationals aged ≥18 years, which reported 18.6% physician-diagnosed PCOS prevalence [43]. In contrast, other UAE studies among university students aged 15–25 years and adults aged ≥25 years reported 13.0% to 27.6% PCOS prevalence [12,44,45]. The differences in diagnostic criteria, sample characteristics or age inclusion criteria, and sampling designs may contribute to the wide range of PCOS prevalence observed in the UAE. Middle Easterners are known to have a higher PCOS prevalence compared to other ethnicities [46], ranging from 6.1% to 16.0% depending on the diagnostic criteria of PCOS [47]. A previous study found certain factors promoting PCOS pathogenesis pertinent to the UAE population, including vitamin D deficiency and obesity [4,46]. Our study also showed that women with PCOS had a higher percentage of being obese, compared to those without PCOS (Table 1). Obesity is known to be a risk factor and a manifestation of PCOS [46]. A current study found that 39.6% of Emirati women are classified as obese [48]. This phenomenon contributes to an increasing rate of PCOS in this country since obesity increases PCOS susceptibility [46].

We found that women with PCOS were 55% more likely to frequently take a course of antibiotics in the past year compared to those without PCOS. In addition, we observed more women with PCOS reported as “frequent users” of antibiotics (those who had taken antibiotics at least three times in the past year), compared to those without PCOS (Figure 2). A previous study reported that women with PCOS had a higher susceptibility to infections than those without PCOS [11,24,25]. PCOS is a pro-inflammatory endocrine disorder and the altered immune features in those affected by PCOS may explain their susceptibility to infections [23], hence more frequent users of antibiotics among women with PCOS than in the control group. On the one hand, we noted that women with PCOS had a higher percentage of reported regular medication use (Table 1), compared to those without PCOS. Our results are in agreement with previous studies that also found similar findings and highlighted that women with PCOS are burdened with medication use and comorbidity [11]. This study reported that women with PCOS are known to take more dermatological and hormonal medications compared to those without PCOS [11]. On the other hand, we also found that more women with PCOS reported being in poorer health, compared to those without PCOS. A previous cohort study in the UK revealed that any comorbidity increased antibiotic prescriptions by 62% among women in primary care settings [49]. Hence, there is a possibility that the increased antibiotic use among those with PCOS in our study was related to their comorbidities. However, we addressed this possibility by performing a sensitivity analysis evaluating only those who reported diabetes, cholesterol, or high blood pressure (Table 3), and we found similar results to that of the main regression analysis (Table 2). With higher magnitudes of associations observed in all strata, this may suggest the possibility of comorbidities modifying the associations between PCOS-related status and antibiotic use. Addressing inappropriate antibiotic use in individuals with PCOS could contribute to broader efforts to combat antibiotic resistance. Future antibiotic resistome studies to identify existing mechanisms of the resistance [50], especially linking them to certain conditions such as PCOS, are needed to address existing antibiotic threats more effectively.

In the PCOS treatment stratification, we found that women being treated for PCOS and those without treatment had a significantly increased likelihood of more frequently taking a course of antibiotics in the past year compared to women without PCOS. We found that those without PCOS treatment had a 46% increased likelihood of more frequently taking a course of antibiotics in the past years compared to those without PCOS. In our study, two-thirds of those with PCOS reported not being treated for PCOS. We have a similar finding to a Korean study that revealed two-thirds of women with PCOS were untreated, including without regular exercise as the basic form of PCOS management [51]. However, a higher magnitude of association was observed in the treated PCOS group with a 70% increased likelihood of more frequently taking a course of antibiotics in the past year (Table 2). To provide further evidence to confirm this observation, we also found a higher proportion of frequent antibiotic users among women being treated for PCOS, compared to those without treatment (Figure 2). This finding raised an important question of whether antibiotic prescription is an integral part of PCOS treatment. PCOS treatment is rarely mono-therapeutic, depending on PCOS signs and symptoms; therefore, PCOS treatment must be personalized to meet each patient’s needs [9]. Existing treatment modalities for PCOS include surgery, contraceptives, as well as pharmacological and non-pharmacological interventions through improved nutritional or diet and physical activity (lifestyle changes to lose weight) [9,32,45]. The possible links between PCOS and antibiotic use can be explained as follows. The intestinal flora in individuals with PCOS differ from those without PCOS, with a higher abundance of certain gut microbiota that promote PCOS [13,14]. A previous study on human subjects and animal models revealed that removing these microbiota through antibiotics improved PCOS symptoms due to decreased serum testosterone levels [14]. Women with PCOS are known to have elevated testosterone levels [5]; therefore, decreasing testosterone levels has been shown to have positive effects on women with PCOS [4]. Future studies investigating the pattern of antibiotic prescriptions specifically used for PCOS management are recommended to better reveal the efficacy of antibiotics in treating PCOS symptoms.

We stratified the analysis by the age at PCOS diagnosis to better understand two distinct PCOS phenotypes (diagnosed earlier versus later in life) and their associations with antibiotic use. We found that those diagnosed with PCOS before 25 years of age were 60% more likely to frequently take a course of antibiotics in the past year compared to those without PCOS (Table 2). Meanwhile, a marginal association was observed between those diagnosed with PCOS at ≥25 years of age, compared to their healthier counterparts. We had 11% missing values for the age at PCOS and lower counts of those with older adults PCOS, and this may contribute to the observed marginal association of PCOS diagnosed at ≥25 years of age with antibiotic use. Nevertheless, the magnitude and direction of the association in this category were consistent with the findings in the younger PCOS category (diagnosed with PCOS at <25 years of age). Those with PCOS diagnosed at ≥25 years of age had a higher proportion of reporting being frequent antibiotic users, compared to those diagnosed before 25 years (Figure 2). Our findings highlighted two distinct PCOS phenotypes, as previous studies have suggested [52,53,54]. The expression of PCOS diagnosed in earlier adulthood differs in clinical and endocrinological features from that of PCOS diagnosed in later adulthood [52]. These differences in clinical and biochemical presentations of PCOS with age lead to the age-related diagnostic criteria of PCOS [54]. Previous studies have documented higher androgen levels in younger PCOS compared to older PCOS [55,56], implying elevated testosterone levels in the younger PCOS group. This may, to some extent, explain our findings, as we observed the strongest magnitude of the association between PCOS and antibiotic use among those with PCOS diagnosed in earlier life. Further, in the UAE context, it has been reported that the most prevalent PCOS symptom among women aged 18–25 years is acne [45]. Oral antibiotics have been used to treat acne vulgaris, a type of acne affecting adolescents and young adults [57], and may partially explain our finding of a stronger and more significant association between those with PCOS diagnosed before the age of 25 years and antibiotic use. In this study, we did not have any information on certain types of antibiotics and whether their prescription was indicated to treat PCOS symptoms among women affected by this hormonal disorder. Therefore, the findings in this study should be interpreted carefully. Better studies to confirm our findings, with complete information on the types of antibiotics and the purpose of antibiotic prescriptions among those affected with PCOS are highly recommended.

Lastly, we performed the same regression analysis among only obese participants to address the possibility of obesity mediating the associations between PCOS-related status and antibiotic use in the past year. We found that all significances in the previously observed associations in the PCOS status, PCOS treatment, and the age at PCOS diagnosis groups disappeared if the analyses were restricted only to those with obesity (Table 4). These findings suggested the possibility of obesity mediates the associations between PCOS-related status and antibiotic use. With the greatest decrease in the magnitude of association was found in the PCOS treatment group, suggesting the stronger role of obesity in mediating the association between PCOS treatment and antibiotic use. Obesity is linked to PCOS in many ways, including low-grade inflammation that leads to insulin resistance and metabolic disorders [58]. The exact link between the pro-inflammatory state of obesity and infections leading to antibiotic use is still unclear. However, a compromised immune response and leptin deficiency in obese individuals were suggested as possible mechanisms of the link between frequent antibiotic use among those living with obesity [27,28]. In addition, the pharmacodynamic parameters, such as volume of distribution and clearance in many commonly used antimicrobials, and the rate of metabolism in individuals with obesity differ from those in the normal weight category [59]. Therefore, it is possible that being obese may lead to inadequate antibiotic dosing [27]. With the frequent co-occurence of obesity with PCOS [26], the appropriateness of antibiotic dosing among those with PCOS merits special attention and further investigation.

### Strengths and Limitations

To our knowledge, this is the first population-based study to investigate the associations between PCOS status and antibiotic use with further stratification by PCOS treatment and the age at PCOS diagnosis among the Emirati population. We were able to examine the associations between PCOS-related status and antibiotic use in the past year with clear temporality. We only included PCOS diagnosis at least one year before the survey time to the recall of antibiotic use in the past year, to avoid the overlapping timeline. However, our large sample size made it possible to perform multiple stratification analyses. We were also able to control for important confounding factors based on the literature. Our study has several limitations. Our findings are specific to Emirati females aged 18–62 years and may not be generalizable to other populations. We conducted a cross-sectional sectional study; therefore, we could establish associations but not causation. Next, the diagnosis of PCOS in our study was based on self-reports, raising the concern of accuracy and diagnosis bias. However, a meta-analysis study found that self-reported PCOS was consistent with diagnosis using the Rotterdam or other criteria [60]. To better address the recall error and cohort effect, we adjusted for age in the adjusted model. In addition, this study might also be prone to possible selection bias due to the convenience sampling design that we used. However, we increased the representativeness of our sample through recruitment in multiple centers across the UAE. Next, there was also a possibility that those with severe PCOS or comorbidity and acute infections might not be able to participate in this study due to their limiting conditions. Further, we do not have any information on the types of antibiotics, the duration of use, or the quantity or total dosage for each course. Lastly, residual confounding factors are possible due to the observational nature of our study, including PCOS severity as it has been found to be associated with PCOS-related microbial pathways [61].

## 5. Conclusions

Our study suggests that PCOS was associated with increased use of antibiotics among Emirati women, especially among women treated for PCOS or without treatment and those diagnosed before the age of 25 years. Understanding the frequent antibiotic use susceptibility among those with PCOS may improve antibiotic use surveillance and promote antibiotic stewardship in these at-risk individuals. Future studies to evaluate the appropriateness of antibiotic prescriptions among those with PCOS merits further investigations, particularly within the context of the global problem of inappropriate antibiotic use (misuse or overuse) that may lead to antibiotic resistance. Lastly, further longitudinal or experimental studies would be needed to establish a causal relationship to better address the link between PCOS and antibiotic use.

## Figures and Tables

**Table 1:** Demographic characteristics and health profiles of study participants based on PCOS status.

Demographic Characteristics and Health Profiles	Without PCOS (*n* = 1701, 82.5%)	With PCOS (*n* = 362, 17.6%)
Demographic characteristics		
Age, median (IQR range), year	22.0 (20–31)	28.5 (23–36)
Urbanicity		
Rural or non-urban areas	240 (14.1)	36 (9.9)
Urban areas	1461 (85.9)	326 (90.1)
Education		
12 years of schooling or below	785 (46.2)	121 (33.4)
Above 12 years of schooling	916 (53.9)	241 (66.6)
Health profiles		
BMI, median (IQR range), kg/m^2^	23.9 (20.4–28.9)	26.6 (23.0–30.9)
BMI categories, without missing category, reported		
Normal BMI or below (<25 kg/m^2^)	835 (49.1)	125 (34.5)
Overweight (25 kg/m^2^ to <30 kg/m^2^)	335 (19.7)	85 (23.5)
Obese (30 kg/m^2^ or above)	313 (18.4)	99 (27.4)
Overall health status		
Poor or fair	426 (25.0)	101 (27.9)
Excellent or good	1275 (75.0)	261 (72.1)
Smoking, without missing category reported		
Never	1410 (82.9)	280 (77.4)
Ever	148 (8.7)	48 (13.3)
Regular medication use *		
No	702 (41.3)	115 (31.8)
Yes	999 (58.7)	247 (68.2)

* Consumed at least one of the following medications: high blood sugar medication, high cholesterol medication, high blood pressure medication, aspirin, paracetamol, regular prescription medication, vitamins, or other supplementations.

**Table 2:** Crude and adjusted ordinal logistic regression analysis of the associations between antibiotic use in the past year and PCOS status among total participants (main analysis), as well as PCOS treatment status and the age at PCOS diagnosis (stratified analysis). Reference group in all analyses: women without PCOS history.

	Antibiotic Use in the Past Year			
	Crude Model	*p*-Value	Adjusted Model *	*p*-Value
	OR (95% CI)		OR (95% CI)	
**Main analysis**				
Without PCOS (*n* = 1701)	(Reference)		(Reference)	
With PCOS (*n* = 362)	**1.65 [1.34–2.02]**	**<0.001**	**1.55 [1.26–1.90]**	**<0.001**
**PCOS-treatment-stratified models**				
*No*				
Without PCOS (*n* = 1701)	(Reference)		(Reference)	
Without PCOS treatment (*n* = 242)	**1.57 [1.23–1.99]**	**<0.001**	**1.46 [1.14–1.87]**	**0.003**
*Yes*				
Without PCOS (*n* = 1701)	(Reference)		(Reference)	
Being treated for PCOS (*n* = 120)	**1.81 [1.30–2.52]**	**<0.001**	**1.70 [1.22–2.38]**	**0.002**
**Age-of-PCOS-diagnosis-stratified models**				
*<25 years of age*				
Without PCOS (*n* = 1701)	(Reference)		(Reference)	
PCOS diagnosed at <25 years (*n* = 211)	**1.65 [1.28–2.13]**	**<0.001**	**1.60 [1.24–2.07]**	**<0.001**
*>25 years of age*				
Without PCOS (*n* = 1701)	(Reference)		(Reference)	
PCOS diagnosed at ≥25 years (*n* = 108)	**1.66 [1.17–2.37]**	**0.005**	1.44 [0.99–2.10]	0.055

* Adjusted for age (continuous), urbanicity (rural/urban), education level (≤12 years/>12 years), BMI category (normal or below; overweight/obese), overall health status (excellent, good/fair, or poor), smoking (no/yes), and regular medication use (no/yes). Statistical significance at the 0.05 level is marked in **bold**.

**Table 3:** Crude and adjusted ordinal logistic regression analysis of the associations between antibiotic use in the past year and PCOS status, restricted only among participants with a history of diabetes, high cholesterol, and high blood pressure). Reference group: women with at least one of comorbidity of diabetes, high cholesterol, or high blood pressure and with no PCOS history, *n* = 470.

	Antibiotic Use in the Past Year			
	Crude Model	*p*-Value	Adjusted Model *	*p*-Value
	OR (95% CI)		OR (95% CI)	
**Main analysis**				
Without PCOS (*n* = 338)	(Reference)		(Reference)	
With PCOS (*n* = 132)	**1.78 [1.24–2.55]**	**0.002**	**1.69 [1.17–2.46]**	**0.006**
**PCOS-treatment-stratified models**				
*No*				
Without PCOS (*n* = 338)	(Reference)		(Reference)	
Without PCOS treatment (*n* = 88)	**1.68 [1.10–2.57]**	**0.017**	**1.59 [1.03–2.46]**	**0.038**
*Yes*				
Without PCOS (*n* = 338)	(Reference)		(Reference)	
Being treated for PCOS (*n* = 44)	**1.95 [1.12–3.39]**	**0.018**	**1.87 [1.06–3.30]**	**0.031**
**Age-at-PCOS-diagnosis-stratified models**				
*<25 years of age*				
Without PCOS (*n* = 338)	(Reference)		(Reference)	
PCOS diagnosed at <25 years (*n* = 68)	**2.08 [1.32–3.28]**	**0.002**	**2.09 [1.30–3.36]**	**0.002**
*>25 years of age*				
Without PCOS (*n* = 338)	(Reference)		(Reference)	
PCOS diagnosed at ≥25 year (*n* = 47)	1.51 [0.86–2.64]	0.154	1.31 [0.73–2.36]	0.363

* Adjusted for age (continuous), urbanicity (rural/urban), education levels (≤12 years/>12 years), BMI category (normal or below/overweight/obese), overall health status (excellent or good/fair or poor), smoking (no/yes), regular medication use (no/yes). Statistically significance at the 0.05 is marked in **bold**.

**Table 4:** Crude and adjusted ordinal logistic regression analysis of the associations between antibiotic use in the past year and PCOS status, restricted only among obese participants, in the main analysis and stratified by PCOS treatment status and age at PCOS diagnosis (reference group: obese women without PCOS history); *n* = 412.

	Antibiotic Use in the Past Year			
	Crude Model	*p*-Value	Adjusted Model *	*p*-Value
	OR (95% CI)		OR (95% CI)	
**Main analysis**				
Without PCOS (*n* = 313)	(Reference)		(Reference)	
With PCOS (*n* = 99)	1.50 [0.99–2.27]	0.054	1.42 [0.93–2.17]	0.109
**PCOS-treatment-stratified models**				
*No*				
Without PCOS (*n* = 313)	(Reference)		(Reference)	
Without PCOS treatment (*n* = 71)	**1.61 [1.01–2.58]**	**0.050**	1.50 [0.92–2.44]	0.103
*Yes*				
Without PCOS (*n* = 313)	(Reference)		(Reference)	
Being treated for PCOS (*n* = 28)	1.28 [0.63–2.63]	0.494	1.19 [0.57–2.46]	0.641
**Age-at-PCOS-diagnosis-stratified models**				
*<25 years of age*				
Without PCOS (*n* = 313)	(Reference)		(Reference)	
PCOS diagnosed at <25 years (*n* =48)	1.28 [0.74–2.21]	0.375	1.24 [0.71–2.17]	0.446
*>25 years of age*				
Without PCOS (*n* = 313)	(Reference)		(Reference)	
PCOS diagnosed at ≥25 years (*n* = 39)	1.76 [0.94–3.29]	0.076	1.70 [0.87–3.32]	0.119

* Adjusted for age (continuous), urbanicity (rural/urban), education level (≤12 years/>12 years), BMI level (continuous), overall health status (excellent or good/fair or poor), smoking (no/yes), and regular medication use (no/yes). Statistical significance at the 0.05 level is marked in **bold**.

## Data Availability

The datasets used and/or analyzed during the current study are available from the principle investigators of the UAE Healthy Future Study on reasonable request.
